# Artificial Intelligence-Driven Food Safety: Decoding Gut Microbiota-Mediated Health Effects of Non-Microbial Contaminants

**DOI:** 10.3390/foods15010022

**Published:** 2025-12-22

**Authors:** Ruizhe Xue, Xinyue Zong, Xiaoyu Jiang, Guanghui You, Yongping Wei, Bingbing Guo

**Affiliations:** Beijing Key Laboratory of Environmental and Viral Oncology, College of Chemistry and Life Science, Beijing University of Technology, Beijing 100124, China

**Keywords:** non-microbial food contaminants, gut microbiota, artificial intelligence, microbiome toxicology

## Abstract

A wide range of non-microbial contaminants—such as heavy metals, pesticide residues, antibiotics, as well emerging foodborne contaminants like micro- and nanoplastics and persistent organic pollutants—can enter the human body through daily diet and exert subtle yet chronic effects that are increasingly recognized to be gut microbiota-dependent. However, the relationships among multi-contaminant exposure profiles, dynamic microbial community structures, microbial metabolites, and diverse clinical or subclinical phenotypes are highly non-linear and multidimensional, posing major challenges to traditional analytical approaches. Artificial intelligence (AI) is emerging as a powerful tool to untangle the complex interactions between foodborne non-microbial contaminants, the gut microbiota, and host health. This review synthesizes current knowledge on how key classes of non-microbial food contaminants modulate gut microbial composition and function, and how these alterations, in turn, influence intestinal barrier integrity, immune homeostasis, metabolic regulation, and systemic disease risk. We then highlight recent advances in the application of AI techniques, including machine learning (ML), deep learning (DL), and network-based methods, to integrate multi-omics and exposure data, identify microbiota and metabolite signatures of specific contaminants, and infer potential causal pathways within “contaminant–microbiota–host” axes. Finally, we discuss current limitations, such as data heterogeneity, small-sample bias, and interpretability gaps, and propose future directions for building standardized datasets, explainable AI frameworks, and human-relevant experimental validation pipelines. Overall, AI-enabled analysis offers a promising avenue to refine food safety risk assessment, support precision nutrition strategies, and develop microbiota-targeted interventions against non-microbial food contaminants.

## 1. Introduction

Food safety is undergoing a profound paradigm shift from acute, pathogen-centered hazards to the more insidious challenge of chronic exposure to low-dose chemical contaminants in the diet. Beyond classical microbial risks, a wide spectrum of non-microbial contaminants, including pesticide residues, heavy metals, antibiotics, processing-derived neo-formed compounds, and micro- and nanoplastics, can enter the human body through daily foods [1]. These contaminants, therefore, could pose severe threats to human health. Increasingly, such health effects are recognized as being tightly intertwined with the structure and function of the gut microbiota, which acts as both a target and a mediator of chemical exposure. For example, heavy metals such as cadmium and arsenic decreased the abundance of beneficial bacteria while promoting the growth of resistant strains [2]. Micro- and nanoplastics can reshape microbial niches through physical adsorption, formation of biofilms, and the release of chemical additives [3]. Perturbations in microbial community composition and function (dysbiosis) have been linked to a range of disorders, including inflammatory bowel disease, metabolic syndrome, allergy, and neurobehavioral alterations [4]. Non-microbial food contaminants can directly or indirectly modulate gut microbial ecology: they may inhibit or enrich specific taxa, alter microbial metabolic pathways, change levels of short-chain fatty acids and other bioactive metabolites, and compromise mucus or epithelial barriers [5]. Conversely, microbiota can transform several contaminants into more or less toxic derivatives, thereby reshaping exposure profiles and influencing systemic distribution and organ-specific toxicity [5]. This bidirectional interaction forms a “contaminant–microbiota–host” axis that complicates conventional risk assessment paradigms. Methodologically, decoding this axis is challenging. Real-world dietary exposure involves complex mixtures of contaminants with correlated occurrence patterns and time-varying doses. On the biological side, high-throughput sequencing, metabolomics, and other omics platforms generate ultra-high-dimensional data capturing microbial taxa, genes, pathways, and metabolites, alongside rich host phenotyping. The resulting datasets are typically characterized by small sample sizes relative to the feature space, strong collinearity, sparsity, and non-linear interactions. Traditional statistical models, which are designed for low-dimensional, independent variables and largely linear associations, struggle to capture such complexity, especially when the goal is not only association testing but also prediction, feature prioritization, and mechanistic hypothesis generation.

Artificial intelligence (AI), encompassing machine learning (ML), deep learning (DL), and network-based approaches, offers a promising toolkit to address these challenges. In toxicology and microbiome research, AI has already been used to classify exposure patterns, predict toxicity endpoints, cluster microbiome configurations, and integrate multi-omics layers [6,7]. When applied to food safety, AI can help move from single-contaminant, single-endpoint analyses toward a systems-level view of how multi-contaminant exposures shape microbiota configurations and, through them, influence host health. Supervised learning models, e.g., random forests (RFs), Support Vector Machines (SVMs), gradient boosting decision trees, can identify microbial and metabolic signatures associated with specific contaminants or contaminant mixtures; unsupervised and representation-learning methods can uncover latent structures in joint exposure–microbiome–phenotype spaces; and network and causal discovery algorithms can prioritize putative mechanistic pathways and intervention targets [8,9]. Importantly, emerging explainable AI (XAI) techniques provide new opportunities to translate complex models into biologically interpretable insights that can inform regulation and intervention design [10]. Furthermore, the applications of AI in this field also include the automated and scalable processing of large datasets from metagenomic sequencing, metabolomics, exposomics, etc. [11]; identifying hidden combinatorial patterns of features within pollutant exposure under multi-variable, multi-factor scenarios, which traditional methods may fail to detect [12]; using XAI (e.g., SHapley Additive exPlanations, SHAP, and Local Interpretable Model-agnostic Explanations, LIME) to connect AI’s predictions with specific biological characteristics, ensuring accuracy while providing appropriate explanations [13]; and the potential for AI development to transform scientific research in risk prediction, assessment, and even personalized prevention and treatment.

Against this backdrop, there is a growing need to systematically synthesize how AI can be leveraged to understand and manage gut microbiota-mediated health risks arising from non-microbial food contaminants. This review summarizes current evidence on how major classes of non-microbial food contaminants interact with the gut microbiota and, via this interaction, modulate intestinal and systemic health outcomes. Then, we outline the state of the art in AI applications along the “contaminant–microbiota–host” continuum, covering exposure pattern recognition, multi-omics integration, risk prediction, and the in silico identification of protective dietary or microbiota-targeted interventions. Based on typical case studies, we highlight the progress and innovations in AI applications for assessing the effects of microplastics exposure and predicting health risks. Finally, this review critically discusses the key challenges, including data heterogeneity, the interpretability of AI models and the practice translation. This review provides novel insights into the use of AI for assessing the risks of non-microbial food contaminants on gut microbiota-mediated health effects.

## 2. Non-Microbial Chemical Contaminants in Food Chain and Their Interactions with Gut Microbiota

### 2.1. Micro- (Nano-)Plastics in Food and Their Interactions with Gut Microbiota

Microplastics (MPs) are generally defined as plastic particles smaller than 5 mm (nanoplastics refer to those less than 1 μm), which are regarded as emerging pollutants of global concern [14]. Their chemical compositions are diverse and mainly include polyethylene, polypropylene, and polystyrene, as well as polyethylene terephthalate, and polyvinyl chloride, among others [15,16]. Over time, these particles are transported through air, soil, and water, eventually entering the food chain. They have been detected in a wide range of food products, including seafood (especially filter-feeding shellfish and small fish), table salt, drinking water, bottled beverages, honey, sugar, and even fresh produce [17,18,19]. Recently, with the rapid pace of life and the increasing consumption of takeaway food, the packaging of these foods has become a significant source of microplastics in the human body [20]. As calculated, an adult man consumes 142 microplastic particles daily, equating to 51,814 ± 8172 particles per year, primarily from drinking water and food [19]. For children, the median daily intake of microplastics from food is 553, which would accumulate to 8.32 × 10^3^ particles by the age of 18 [21].

**Figure 1:**
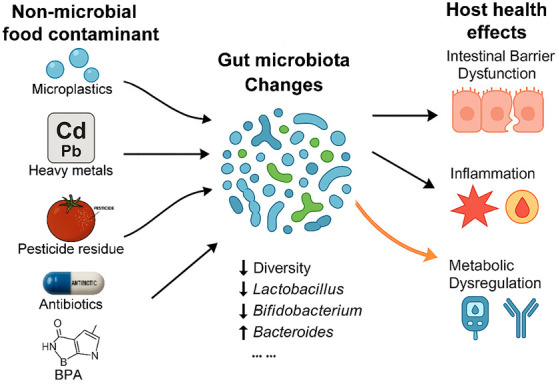
The interaction between non-microbial food contaminants and gut microbiota.

Once microplastics enter the human body through various means, they accumulate primarily in the digestive system, especially in the gut. In vitro studies have shown that microplastics can damage human health by affecting the gut microbiota, inducing oxidative stress, DNA damage, inflammation, genotoxicity, cell membrane damage, and apoptosis of intestinal epithelial cells [22,23]. Animal models have demonstrated that after mice ingest microplastics commonly found in disposable packaging and foam containers, the composition of their gut microbiota changes, marked by a decrease in microbial diversity and a reduction in beneficial bacteria (Figure 1). This leads to metabolic disruption, damage to the intestinal mucosal barrier, and the infiltration of a toxin called LPS into the portal venous system, which releases inflammatory cytokines such as TNF-α and IL-1β, triggering liver inflammation [24]. Furthermore, disrupted microbiota metabolism reduces the production of short-chain fatty acids (especially butyrate) and affects GLP-1 secretion, indirectly inhibiting insulin signaling and triggering a non-alcoholic fatty liver disease (NAFLD) phenotype [25]. Commonly used plastics like polyethylene, which is found in plastic bags, alter the physicochemical properties of the gut, adsorb lipids and bile salts, disrupt microbial niches, and change the pH and redox potential, leading to a reduction in microbial diversity [26]. Moreover, repeated exposure causes an accumulation of reactive oxygen species (ROS) in the gut and an upregulation of IL-6, damaging the intestinal barrier and further altering the microbiota composition, activating oxidative stress and inflammatory pathways [27]. Additionally, poly(ethylene terephthalate), the main component of synthetic fibers or plastic water bottles, can induce DNA damage and oxidative stress in intestinal epithelial cells, activate NF-κB signaling, and disrupt the microbiota structure, ultimately triggering inflammation [28]. The toxic effects of microplastics are closely related to their physicochemical properties. Particle size is a key factor: smaller microplastics, especially those at the nanoscale (<100 nm), are more likely to penetrate the intestinal epithelial barrier, enter the lymphatic and circulatory systems, and ultimately distribute to distant organs, such as the liver, spleen, and even the brain, leading to systemic effects [29,30]. The surface properties of microplastics are also critical. They can serve as substrates for microbial attachment, promoting the formation of biofilms. Such biofilm formation not only alters the characteristics of the microplastics themselves but may also enrich pathogens and pollutants, and through interactions with the native gut microbiota, disrupt microbial homeostasis [31]. These complex pollutant–microbiota–host interactions present potential variables for future AI modeling.

### 2.2. Heavy Metal Contamination in Food and Gut Microbiota Dysbiosis

Among non-microbial contaminants in the food chain, heavy metals represent one of the most widespread and well-characterized classes of toxicants. Heavy metals such as cadmium (Cd), lead (Pb), mercury (Hg) and arsenic (As) can enter foods through contaminated soil and irrigation water, atmospheric deposition, industrial emissions, feed additives, and certain food processing practices. They are frequently detected in cereals, vegetables, rice, seafood, meat products, and drinking water, often at low but chronic exposure levels [32,33]. Unlike many organic pollutants, heavy metals are non-degradable and can bioaccumulate along the food chain, leading to long-term retention in human tissues after ingestion. Traditionally, their health risks have been framed in terms of direct systemic toxicity, such as nephrotoxicity, neurotoxicity, and carcinogenicity, but accumulating evidence indicates that the gut microbiota is a critical early target and mediator of metal-induced health effects [34].

Experimental and epidemiological studies have shown that dietary exposure to heavy metals can profoundly reshape gut microbial ecology (Figure 1). For instance, Cd exposure leads to a significant reduction in both α-diversity and β-diversity of the gut microbiota, with a marked decrease in beneficial bacteria such as *Lactobacilli* and *Bifidobacteria*, while the relative abundance of *Firmicutes* and *Bacteroidetes* increases [35]. Similar to microplastics, Cd can activate the NF-κB pathway, induce ROS production, and upregulate the expression of pro-inflammatory cytokines, such as TNF-α, IL-1β, and IL-6 [34]. Additionally, Cd exposure can reduce the expression of tight junction proteins, such as ZO-1, Claudin-1, and Occludin, ultimately compromising intestinal barrier integrity [34]. Hg disrupts the gut microbiota through several mechanisms, with its primary effects involving changes in community structure, microbial dysfunction, and synergistic barrier damage, particularly with inorganic (IHg) and methylmercury (MeHg) forms [36]. Both IHg and MeHg increase the abundance of pathogenic bacteria like *Staphylococcus*, *Streptococcus*, *Clostridium*, and *Spirochaeta*, while reducing beneficial bacteria such as *Lactobacilli* and *Bifidobacteria*, predisposing to inflammation [37]. In addition to lowering beneficial bacteria, Hg exposure also disrupts the metabolism of several neurotransmitters and their precursors, including GABA, serotonin, dopamine, and tyrosine, thereby impairing gut–brain communication and immune regulation [38]. As mentioned earlier, the gut microbiota also provides protective functions against Cd toxicity. Probiotic bacteria, such as *Lactobacillus* and *Bifidobacterium*, can actively adsorb Cd, mitigating its toxic effects [39]. Thus, the gut microbiota serves as both a target for Cd toxicity and a regulator of its effects. Furthermore, microbes in the gut express genes like hgcAB (which methylates IHg) and merA (which demethylates MeHg). Under Hg exposure, the abundance of these microbes increases, disturbing microbial homeostasis and impairing the stability of the detoxification processes [40]. However, the exact interaction between heavy metal exposure and gut microbiota has not been systematically studied.

### 2.3. Pesticide Residues in the Food Supply and Microbiota-Mediated Health Effects

Another major category of non-microbial contaminants in the food supply is pesticide residues, including insecticides, herbicides, fungicides, and rodenticides that are widely applied in modern agricultural production. Residues of organophosphates, carbamates, pyrethroids, triazole fungicides, glyphosate and its metabolites, among others, are frequently detected in fruits, vegetables, grains, tea, and processed foods, sometimes as complex mixtures rather than single compounds. Among all organophosphates (OPs), triazophos, isocarbophos, chlorpyrifos, diazinon, formothion, and phosphates are the most frequently reported in fruits, vegetables, and tea in China [41]. In a recent report by the European Food Safety Authority, chlorpyrifos residues were detected in dry beans, pears, and rice, while triazophos was found in rice [42]. Although current maximum residue limits (MRLs) are set to protect consumers from acute toxicity, real-world dietary exposure tends to be chronic and low-dose, raising concerns about subtle, long-term effects such as endocrine disruption, immunomodulation, and metabolic disturbances. Increasingly, the gut microbiota is being recognized as both a sensitive sensor of pesticide exposure and a potential mediator of downstream health outcomes.

Accumulating evidence from animal models and human studies indicates that pesticide residues can induce marked alterations in gut microbial community structure and function (Figure 1) [43]. Specific pesticides have been associated with decreased microbial richness and diversity, shifts in microbiota composition, depletion of beneficial genera involved in short-chain fatty acid production, and enrichment of taxa that harbor xenobiotic-degrading enzymes or antibiotic resistance determinants. For example, a study confirmed that organophosphates significantly affected the gut microbiota in mice and zebrafish [44]. Commonly used pesticides promoted endotoxin-producing bacteria, increased *Firmicutes*/*Bacteroidetes*, and enhanced obesity-related flora characteristics [44]. These compositional changes are often accompanied by functional reprogramming, including altered carbohydrate and lipid metabolism, disrupted bile acid and tryptophan pathways, increased production of pro-inflammatory mediators, and enhanced oxidative stress [45]. Such pesticide-induced gut microbiota dysbiosis may compromise intestinal barrier integrity, aggravate low-grade inflammation, and modulate systemic metabolic and neuroendocrine signaling, thereby linking dietary pesticide exposure to outcomes such as obesity, insulin resistance, allergic diseases, and neurobehavioral alterations. In this context, decoding how specific pesticide mixtures reshape the “pesticide–microbiota–host” axis has become an important frontier for both food safety assessment and microbiota-targeted prevention strategies.

### 2.4. Antibiotic and Veterinary Drug Residues in Food and the Gut Resistome

Antibiotics and other veterinary pharmaceuticals represent a distinct and highly microbiota-relevant class of foodborne contaminants. In intensive livestock and aquaculture systems, antimicrobials are widely used not only for therapeutic purposes but also for metaphylaxis, prophylaxis, and, in some regions, growth promotion. Inadequate withdrawal times, off-label use, and environmental contamination of feed and water can result in residues of tetracyclines, β-lactams, macrolides, sulfonamides, fluoroquinolones, and antiparasitic agents in meat, milk, eggs, and aquaculture products, often at low but chronic exposure levels [46,47,48]. While regulatory maximum residue limits are intended to prevent acute toxicity and overt antimicrobial effects, mounting evidence suggests that sub-inhibitory concentrations of these compounds can still exert biologically meaningful pressure on the human gut microbiota (Figure 1).

Dietary exposure to antibiotic and veterinary drug residues can directly select for resistant taxa, expand the gut resistome, and destabilize the ecological balance of the intestinal community [49]. Animal and human studies have reported reduced overall microbial diversity, depletion of beneficial commensals involved in short-chain fatty acid production and mucosal homeostasis, and enrichment of opportunistic or pathobiont species carrying multiple antibiotic resistance genes [50,51]. These shifts are accompanied by increased horizontal gene transfer, altered metabolic networks, and impaired colonization resistance against enteric pathogens. Such residue-induced microbiota dysbiosis may contribute to low-grade inflammation, immune dysregulation, and metabolic disturbances, and can interact with other foodborne contaminants by modifying xenobiotic metabolism and barrier integrity. In this context, antibiotic and veterinary drug residues in food are not only a traditional antimicrobial resistance (AMR) concern, but also a key entry point for AI-driven, microbiota-centered analyses of how chronic low-dose chemical exposures reshape the “contaminant–microbiota–host” axis.

### 2.5. Persistent Organic Pollutants (POPs) and the “POPs–Microbiota–Host” Axis

Persistent organic pollutants (POPs) constitute another important group of non-microbial contaminants of concern in the food chain. Classical POPs, such as polychlorinated biphenyls (PCBs), dioxins and furans, organochlorine pesticides (e.g., DDT and its metabolites), polybrominated diphenyl ethers (PBDEs), and certain polycyclic aromatic hydrocarbons (PAHs), together with more recently highlighted compounds, such as some per- and polyfluoroalkyl substances (PFAS), are characterized by high lipophilicity, environmental persistence, and strong bioaccumulation potential [52]. For instance, PCBs (0.1–0.8 µg kg^−1^) and PFAS (0.05–0.3 µg kg^−1^) were largely found in seafood [52]. These POPs can enter foods via contaminated soil, water, and air, as well as through feed and food processing, and are especially enriched in fat-rich animal products, such as meat, dairy, eggs, and certain fish. Even when present at low concentrations, their long half-lives and tendency to biomagnify along the food chain can lead to significant body burdens over time, which have traditionally been linked to endocrine disruption, immunotoxicity, carcinogenicity, and adverse developmental outcomes. In particular, bisphenol A (BPA), as an endocrine-disrupting chemical, is widely present in everyday products such as plastics, food packaging coatings, and dental composites, leading to widespread human exposure [53].

This extensive exposure has raised concerns about its potential health risks, particularly its effects on the gut microbiota [54] (Figure 1). A rat study using high-throughput 16S rRNA sequencing systematically evaluated the effects of BPA exposure on gut microbiota composition and diversity. Analysis of fecal samples through V3–V4 region sequencing revealed that BPA exposure significantly disrupted the gut microbiota ecosystem in rats and directly affected host immunity, endocrine function, and metabolic homeostasis [55]. For example, one study found that intermittent or continuous oral administration of 500 μg BPA/kg body weight/day in rats affected the metabolic capacity of the gut microbiota [56]. Another study in zebrafish also showed that BPA and its substitutes were negatively correlated with the degree of gut microbiota disruption, indicating that the greater the developmental toxicity of BPA to the host, the stronger its interference with the microbiota [57]. In addition, BPA exposure can lead to gut microbiota dysbiosis, which in turn exacerbates oxidative stress, inflammatory responses, and apoptosis, ultimately causing damage to the small intestine, with the duodenum being the most severely affected, characterized by villus atrophy, epithelial shedding, and mitochondrial swelling [57]. Therefore, decoding the interplay of the “POPs–gut microbiota–health” axis is crucial for exploring effective intervention strategies.

### 2.6. Other Foodborne Chemical Contaminants Affecting the Gut Microbiota

Beyond micro/nanoplastics, heavy metals, pesticides, POPs, and antibiotic residues, a wide range of additional foodborne chemicals can perturb the gut microbiota. For instance, food additives and processing-induced contaminants represent another important group. Emulsifiers, non-caloric artificial sweeteners, and other additives, together with neo-formed compounds generated during high-temperature processing (e.g., acrylamide, heterocyclic aromatic amines, nitrosamines, advanced glycation end products), have been shown to promote microbiota dysbiosis, mucus layer thinning, barrier dysfunction, and low-grade inflammation, linking ultra-processed diets to obesity and cardiometabolic diseases via microbiota-mediated mechanisms [58]. In addition, naturally occurring toxins, such as mycotoxins (e.g., aflatoxins, deoxynivalenol, zearalenone), and selected engineered nanomaterials (e.g., food-grade titanium dioxide) can be biotransformed by, and simultaneously reshape, gut microbial communities [59]. Collectively, these “other” contaminants further expand the spectrum of agents capable of disturbing the contaminant–microbiota–host axis and highlight the need for integrative, multi-contaminant assessments in microbiota-centered food safety research.

### 2.7. Co-Exposure and Comprehensive Analysis

In real-world settings, humans are rarely exposed to a single contaminant in isolation; instead, they experience chronic, low-dose co-exposure to complex mixtures of heavy metals, pesticide residues, persistent organic pollutants, micro/nanoplastics, and other emerging chemicals through daily diets. These contaminants may share common sources (e.g., contaminated soil or irrigation water), co-occur in the same food items, or act sequentially along the food chain. From the perspective of gut microbiota and host health, such co-exposures can give rise to additive, synergistic, or antagonistic effects, which are fundamentally different from those observed under single-compound exposure paradigms. For example, microplastics may serve as physical carriers that adsorb POPs, metals, or pesticide residues, altering their bioavailability and contact with the intestinal mucosa; metals and pesticides may jointly select for resistant microbial taxa and resistance gene pools; and overlapping pathways of oxidative stress, inflammation, and metabolic disruption can be either amplified or partially compensated depending on the mixture composition and timing of exposure [60]. One study demonstrated that under co-exposure conditions, the combined action of microplastics and 2,6-dichloro-1,4-benzoquinone (DCBQ) might induce stronger immune responses in mice than either pollutant alone [61]; simultaneous presence of microplastics and cadmium (Cd) was more likely to cause intestinal barrier damage than when they were present individually [62].

This complexity poses substantial challenges for conventional food safety assessment, which has historically relied on single-compound toxicological data, default dose-addition models, and relatively simple exposure metrics. In the context of the gut microbiota, co-exposure leads to high-dimensional, intertwined perturbations in microbial taxonomic profiles, functional gene repertoires, and metabolite networks, making it difficult to disentangle which contaminants or combinations are driving specific dysbiosis patterns and health outcomes. Moreover, individual variability in diet, host genetics, baseline microbiota configuration, and life-stage further modulates responses to contaminant mixtures, resulting in heterogeneous and often non-linear exposure–response relationships that are poorly captured by traditional statistical approaches.

## 3. AI Enables the Analysis of Non-Microbial Contaminant–Gut Microbiota–Host Interactions

### 3.1. AI-Assisted Deciphering of Gut Microbiota Changes Induced by Non-Microbial Contaminants

#### 3.1.1. AI for Microbiome Characterization and Dysbiosis Mapping

**Figure 2:**
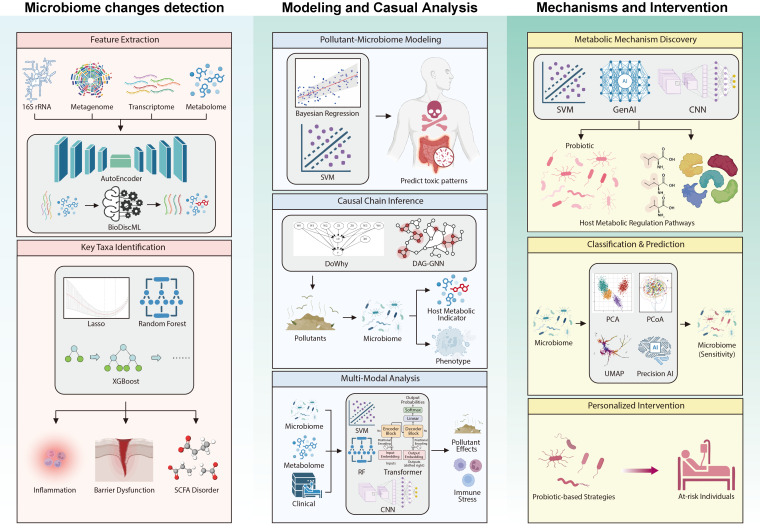
AI assists the study of interactions between non-microbial food contaminants and gut microbiota-mediated health.

AI has become a cornerstone for microbiome characterization [63], including in settings where foodborne contaminants interact with the gut microbiota (Figure 2). Food toxicology and microbiome studies routinely generate multi-layered omics data, including 16S rRNA profiles, shotgun metagenomics, metatranscriptomics, metabolomics, and metaproteomics, which are high-dimensional, sparse, noisy, and strongly correlated. These properties make it difficult to interpret contaminant–microbiota–host interactions using conventional low-dimensional, linear models and create a pressing need for robust AI-based dimensionality reduction and feature extraction strategies.

The first class of methods relies on unsupervised dimensionality reduction and visualization techniques, such as principal component analysis (PCA), principal coordinates analysis (PCoA), t-distributed stochastic neighbor embedding (t-SNE), and uniform manifold approximation and projection (UMAP) [64]. Applied to high-dimensional, sparse microbiome data, these approaches compress the feature space while preserving local and/or global sample relationships, thereby enabling intuitive visualization of dysbiosis patterns. They help researchers identify clusters of samples with similar microbiota configurations, delineate microbiome subtypes associated with specific contaminant exposure patterns, and highlight potential microbial targets for intervention. For example, UMAP has been used to clearly separate patient and control groups in irritable bowel syndrome (IBS) [65] and non-alcoholic fatty liver disease (NAFLD) [66] cohorts, revealing subtle yet systematic community-level shifts that might be overlooked by univariate analyses.

Beyond classical manifold learning, AutoEncoder represents a typical neural network-based approach for non-linear dimensionality reduction. It can compress high-dimensional input data into low-dimensional latent representation and then attempts to reconstruct the original input from this compressed code. In doing so, AutoEncoder can learn complex non-linear mappings and capture higher-order interactions in sparse microbiome data. In the context of contaminant–gut microbiota research, AutoEncoder can be used to derive latent microbiota patterns associated with specific contaminant mixtures, build classifiers that predict disease states (e.g., type 2 diabetes or inflammatory bowel disease) from compressed feature vectors, integrate microbiome with metabolomic or transcriptomic data in a shared latent space, and detect anomalous samples based on high reconstruction error (e.g., technical artifacts or extreme exposure profiles) [67].

Beyond classical manifold learning, AutoEncoder represents a typical neural network-based approach for non-linear dimensionality reduction. It can compress high-dimensional input data into low-dimensional latent representation and then attempts to reconstruct the original input from this compressed code. In doing so, AutoEncoder can learn complex non-linear mappings and capture higher-order interactions in sparse microbiome data. In the context of contaminant–gut microbiota research, AutoEncoder can be used to derive latent microbiota patterns associated with specific contaminant mixtures, build classifiers that predict disease states (e.g., type 2 diabetes or inflammatory bowel disease) from compressed feature vectors, integrate microbiome with metabolomic or transcriptomic data in a shared latent space, and detect anomalous samples based on high reconstruction error (e.g., technical artifacts or extreme exposure profiles) [67].

In clinical settings, BioDiscML, an automated biomarker discovery tool, is designed for high-dimensional omics and clinical data. Its core mechanism integrates a complete ML workflow, including data preprocessing, feature ranking and dimensionality reduction, feature subset selection, model search, and evaluation. The major advantages of BioDiscML lie in its support for multi-source data, both classification and regression tasks, and its ability to enhance efficiency and predictive performance through parallelization and multi-model integration, enabling non-linear dimensionality reduction in data processing. When studying the effects of pollutant exposure on gut microbiota, BioDiscML can be applied to select key molecular biomarkers from multidimensional microbiome, metabolome, and exposome datasets, thereby identifying associative features among contaminants, microbial structural variations, and host health states. In this way, such tools provide powerful computational support for dysbiosis mapping, mechanistic hypothesis generation, and risk prediction in environment-mediated gut microbiota perturbations [68].

#### 3.1.2. AI for Identifying Key Bacterial Genera or Metabolic Pathways Highly Correlated with Contaminant Exposure

One of the central challenges in microbiome-oriented food safety research is to identify bacterial genera or metabolic pathways that are most sensitive to exposure of non-microbial contaminants in food. Ensemble learning methods based on decision trees are widely used in this context because of their robustness to high-dimensional feature spaces, ability to capture non-linear interactions, and relatively high interpretability. These methods train multiple decision trees and aggregate their outputs (e.g., by majority vote or weighted averaging), thereby reducing overfitting and improving generalization. Variable importance scores derived from such models provide a quantitative measure of how strongly each genus or pathway contributes to the prediction, and thus serve as a natural basis for biomarker discovery under dietary exposure scenarios [69].

RF and XGBoost are typical decision tree-based ML algorithms applied in studies of gut microbiota and foodborne chemical contamination. RF builds a large number of decorrelated trees on bootstrapped samples, while XGBoost optimizes an ensemble of gradient-boosted trees with regularization to improve both accuracy and computational efficiency. In the context of non-microbial food contaminants and gut microbiota, RF and XGBoost can be used for identifying key bacterial genera and metabolic pathways. For instance, with RF or XGBoost, relative abundance of genera can be used as input features and pollutant exposure levels or doses as output labels to train a classification model. The variables are ranked by their importance to the classification, helping identify bacterial genera that are most sensitive to pollution, such as *Bacteroides*, *Prevotella*, *Alistipes*, and *Ruminococcus*, which are associated with inflammation or gut barrier function [70]. If KEGG pathway abundances are used as inputs (for example, those predicted by PICRUSt2), ML algorithms such as RF or XGBoost can identify functional modules significantly affected by pollutant exposure, including fatty acid metabolism, aromatic hydrocarbon degradation, retinol metabolism, and the NOD-like receptor signaling pathway. These modules can serve as potential biomarkers or predictive factors for environmental toxicity. For instance, J. Wang et. al. [71] employed an XGBoost-based approach to investigate the metabolic pathways affected by BPA exposure.

Beyond tree-based ensembles, other ML approaches also contribute to feature selection and biomarker discovery in the context of non-microbial food contaminants (Figure 2). Regularized regression models (e.g., LASSO, elastic net), SVM with embedded feature ranking and gradient boosting frameworks can all be used to derive sparse sets of discriminative taxa or pathways while controlling for multicollinearity [72,73]. In more complex settings that integrate microbiome data with metabolome or exposome layers (e.g., dietary chemical profiles, biomarkers of internal exposure), multi-task learning and multi-output models can jointly prioritize features that are predictive of multiple outcomes, such as contaminant burden, intermediate biochemical markers, and clinical endpoints, thereby enriching the biological information content of the selected taxa and pathways [74].

Increasingly, XAI techniques are being combined with these models to refine the identification of key bacterial genera and pathways under foodborne chemical exposure. Methods such as SHAP and LIME can decompose model predictions into contributions from individual features at both global and sample-specific levels [75]. Applications of RF, XGBoost, or DL models, together with SHAP and related tools, allow researchers not only to rank genera or pathways by importance but also to visualize how changes in their abundance across the exposure gradient influence predicted risk or exposure status [76]. This helps distinguish truly informative features from spurious correlations driven by confounding or co-occurrence patterns.

### 3.2. Modeling and Prediction of the Relationship Between Non-Microbial Contaminant and Gut Microbiota

#### 3.2.1. Prediction of Interaction Between Non-Microbial Food Contaminants and Gut Microbiota

The disruption patterns of the gut microbiome and its response to non-microbial food contaminants vary significantly across individuals and contaminants, presenting challenges in identifying the key microbial species and/or metabolites that respond to these contaminants. Several regression models show potential for connecting gut microbiota changes induced by contaminants. For example, Bayesian regression, a classical statistical method, has been used to analyze microbiome data derived from 16S rRNA sequencing or metagenomic sequencing of human fecal or intestinal samples. In such experiments, Bayesian methods can handle high-dimensional count matrices, preventing issues like sequencing depth differences between samples, thereby reducing potential normalization bias and information loss [77]. Furthermore, by incorporating clinical indicators, metabolite levels, and environmental exposure data, Bayesian regression models can estimate the influence of these covariates on microbiome structure, ensuring that confounding factors are appropriately controlled for in the analysis [78,79,80]. Another valuable tool in analyzing non-microbial food contaminants and gut microbiota is Lasso regression, which is particularly effective for high-dimensional data. Lasso regression can shrink regression coefficients of less important variables to zero, performing both variable selection and model fitting. In the context of microbiome analysis, Lasso regression can use Operational Taxonomic Unit (OTU) or Amplicon Sequence Variant (ASV) tables, where microbial abundances are treated as independent variables and pollutant exposure levels or biochemical indicators as dependent variables. The non-zero coefficients in the output represent the core genera most associated with pollutant exposure and toxicity, thereby identifying key microbial targets for understanding foodborne pollutant toxicity [81]. For metabolic pathway selection, functional pathway abundances (such as those predicted by PICRUSt2 or KEGG) can be used as features, with Lasso regression helping to predict pollutant concentrations or oxidative stress levels, offering a more focused interpretation of potential toxicological mechanisms associated with food contaminants [81]. Additionally, SVM is a powerful classification method that has been successfully applied in disease diagnosis, including cancer and metabolic disorders. With the advancement of high-throughput technologies, which have generated vast amounts of data, the classification capabilities of SVM have been continuously expanded, thus broadening its application in the biomedical field. For instance, by employing three ML models (SVM, XGBoost, and MLP) alongside six clinical features and ten types of gut bacteria, the model’s prediction performance was assessed using the area under the receiver operating characteristic curve (AUC), successfully predicting the risk of type 2 diabetes mellitus (T2DM) [82]. This highlights how ML algorithms can be leveraged to identify specific microbial species that are most sensitive to foodborne contaminants, offering insights into their role in mediating health outcomes.

#### 3.2.2. Causal Inference of “Toxicity Causal Chains”

Causal inference models, such as Graph Neural Networks (GNNs), are increasingly used to uncover potential mechanisms linking exposure to foodborne contaminants with microbiome dysbiosis and host health outcomes (Figure 2). By modeling causal relationships, these methods help trace how contaminants disrupt microbial communities and how these disruptions, in turn, influence host phenotypes. One notable example of AI-based causal inference is the DoWhy causal discovery algorithm, which allows researchers to infer the causal chain between pollutant exposure, microbiome alterations, and resulting health effects. In a study examining neonatal jaundice, the DoWhy + EconML library was used to construct a causal graphical model incorporating gut microbiome abundance, bile acid metabolites, serum total bilirubin (TBIL), and disease status [83]. The study performed a full causal inference analysis to evaluate both direct and indirect causal effects between the microbiome and bile acid metabolism, revealing how shifts in microbial populations can affect TBIL levels, thus contributing to the development of neonatal jaundice [83]. This approach not only resolves the issue of “correlation does not imply causation” but also provides an understanding of the microbial mechanisms by which foodborne contaminants mediate disease onset.

Another advanced method in causal modeling is DAG-GNN, a deep generative model that integrates Variational Autoencoders (VAEs) with GNN to learn a Directed Acyclic Graph (DAG) from observed microbiome and environmental data [84]. DAG-GNN enables the automatic discovery of non-linear causal relationships between pollutants, microbiota, and host phenotypes by incorporating acyclicity constraints, ensuring that the resulting graph remains a valid causal model. This model shows potential performance in learning complex dependencies in synthetic datasets and has been successfully applied to microbiome research. This methodology does not rely on pre-specified causal graph structures, allowing for flexible exploration of novel causal relationships and providing a more accurate understanding of pollutant–microbiome interactions.

#### 3.2.3. AI-Driven Multimodal Data Integration

Exposure to non-microbial food contaminants is often accompanied by a diverse array of multimodal data, including urine metabolomics, plasma inflammatory markers, and gut microbiome profiles. These multimodel data types provide a comprehensive view of the biological processes underlying toxicity and disease. The integration of such varied data, coupled with the power of AI, enables a more holistic and panoramic decoding of toxicity mechanisms, advancing both predictive capabilities and mechanistic exploration of the effects of these non-microbial contaminants on human health.

AI techniques, particularly multimodal learning, allow for the integration of microbiome, metabolomics, and clinical data, which collectively enhance the ability to uncover complex and non-linear relationships between microbiota, metabolites, and host phenotypes (Figure 2). For example, combining ML models, such as SVM, RF, and CNN with multimodel datasets has revealed hidden interactions between microbiota and metabolites, as well as microbiota–microbiota interactions that are crucial for predicting disease outcomes [85]. These models have demonstrated increased accuracy in identifying associations between contaminant exposure and gut microbiome dysbiosis, as well as their subsequent impact on health conditions, like inflammation and metabolic disorders [85]. This highlights the utility of AI in not only identifying toxicological effects but also enhancing our understanding of how pollutants disrupt microbial communities.

With the rapid advancement of DL, the potential for accurate and efficient multimodal data integration has grown exponentially. One of the most successful and widely recognized models for this purpose is the Transformer model, originally designed for natural language processing (NLP) and subsequently adapted to handle multimodal data in microbiome research. For instance, a research team at Stanford University combined open-source human gut microbiome data with NLP techniques to create a self-supervised “microbial language model” [86]. This model learned interactions between microbial taxa and identified common composition patterns. By processing multimodal data, such as microbiome composition, clinical features, and contaminant exposure levels, it developed a predictive tool for Irritable Bowel Disease (IBD). Notably, the model demonstrated strong cross-population generalization, effectively predicting health outcomes across diverse populations with varying pollutant exposure profiles [86]. Moreover, Transformer models have been used to predict pathogen presence in poultry farms by integrating microbiome data with farm management practices [87]. This demonstrates the versatility of AI in understanding how environmental factors, including non-microbial food contaminants, influence the microbiome in agricultural settings, highlighting the potential for predictive modeling in food safety management.

### 3.3. AI-Driven Analysis of Metabolic Mechanisms and Gut Microbiota-Targeted Interventions of Non-Microbial Contaminants on Health

#### 3.3.1. Mechanistic Insights of Non-Microbial Contaminants Through Gut Microbiota Interventions in Host Health

AI methodologies, particularly those based on ML, have proven to be instrumental in elucidating the complex mechanisms through which these non-microbial food contaminants exert their toxic effects on the microbiome and, by extension, the host (Figure 2). At the molecular level, AI enables the integration of metagenomic data and antibiotic resistance gene databases, facilitating an understanding of how gut microbes adapt to environmental changes, particularly under antibiotic pressure, and the mechanisms through which they develop resistance [88]. Furthermore, AI can uncover gene network structures associated with resistance, identifying previously unnoticed interaction-based resistance mechanisms [89,90]. Regarding target discovery, AI models, such as SVM, RF, CNN, RNN, GNN, and DeepMicro, can determine potential functional failures and identify associated symptoms by identifying related microbiome features [91,92]. By analyzing a patient’s metabolite profile from urine, blood, or stool, AI can identify which metabolites are most suitable for targeted modulation and make specific recommendations for microbial species combinations. For instance, certain combinations of *Lactobacillus* and *Bifidobacterium* have been found to improve symptoms of adult IBS, based on the correlation between AI-predicted metabolic targets and associated microbiome features [92]. This forms the basis for conducting health risk assessments of foodborne non-biological contaminants.

#### 3.3.2. Prediction of Individualized Toxicity Responses and Identification of Sensitive Contaminants

Predicting individualized toxicity responses to non-microbial food contaminants (e.g., pesticides, heavy metals) is crucial for identifying sensitive populations at higher risk of adverse health outcomes. AI and ML offer powerful tools for personalized risk prediction, integrating diverse data sources, such as microbiome composition, genetics, and clinical indicators. For instance, UMAP visualizations can reveal non-linear relationships between microbial communities in healthy versus exposed individuals, helping identify potential biomarkers for contaminant sensitivity [93]. AI-based supervised learning models (e.g., RF, SVM) can classify individuals into high-risk or low-risk categories based on their microbiome features and exposure history. These models enable the identification of microbial signatures that predict susceptibility to diseases linked to foodborne contaminants, such as IBS or metabolic disorders. Clustering algorithms further refine this process by grouping individuals with similar toxicological profiles, facilitating targeted interventions, like dietary adjustments or probiotic treatments. Ultimately, AI allows for personalized health predictions by combining multi-omics data to uncover microbial biomarkers and predict individual responses to non-microbial pollutants, guiding precise interventions and improving food safety.

Another key challenge in AI-driven research on foodborne non-biological contaminants and gut microbiome is the scarcity of well-characterized human cohorts with detailed data on contaminant exposure and microbiome composition. For example, while studies have identified associations between certain environmental pollutants, such as heavy metals or pesticide residues, and changes in the gut microbiota, most existing cohorts lack both the comprehensive exposure histories and microbiome data required to fully understand these relationships. This limitation hinders the ability to build robust models that can accurately predict health outcomes. AI methods, however, have the potential to bridge this gap by integrating experimental and observational datasets. For example, AI algorithms can combine controlled experimental data on microbial responses to specific contaminants with large-scale observational studies that capture real-world exposure patterns, leading to more comprehensive and generalizable models. Additionally, causal inference frameworks, such as directed acyclic graphs (DAGs) or structural equation modeling (SEM), can help discern the causal pathways between contaminant exposure, microbiome changes, and health outcomes. To further support AI-driven risk assessments, future study designs should focus on longitudinal cohort studies that include both detailed contaminant exposure histories and comprehensive microbiome profiling, as seen in projects like the Human Microbiome Project (HMP) or the Gut Microbiome and Human Health Study.

#### 3.3.3. AI-Assisted Intervention Design for Gut Microbiota Dysbiosis Induced by Non-Microbial Contaminants

The development of AI-assisted interventions leveraging multi-omics data integration offers a promising approach to personalized health management (Figure 2). By combining longitudinal and cross-sectional data, AI enables a more precise understanding of the dynamic relationships between probiotics, the environment, the host, and other microbial communities, thus facilitating more effective interventions. As mentioned above, AI models, especially those based on ML techniques such as CNN and SVM, allows for the identification of probiotic adaptability patterns, linking these to health effects and individual responses to food contaminants. For instance, by using SVM models, researchers have achieved high accuracy (97.77%) in predicting the most suitable probiotic strains based on microbial composition [94]. Additionally, DL algorithms like CNN have been applied to understand protein structure–function relationships, intestinal parasite detection, and bacterial colony morphology, thereby enhancing the functional analysis of probiotics [95]. Furthermore, Natural Language Processing (NLP) can mine literature data and functional gene clusters, while computer vision enables automated phenotypic feature extraction from probiotic images. This integration of AI technologies provides a more comprehensive approach to probiotic research, enabling the design of more targeted and efficient interventions for individuals exposed to non-microbial food contaminants [96]. In addition to these applications, reinforcement learning has shown potential for real-time modulation of the gut microbiota. This AI technique can dynamically adjust probiotic interventions based on individual microbiome profiles, offering highly personalized approaches to health management [97]. GenAI and edge AI technologies enable the analysis of large-scale, real-time data, accelerating the development of personalized probiotic products [92]. Collectively, by integrating individual microbiome profiles with environmental exposure data, AI allows for the creation of tailored therapeutic strategies for designing interventions aimed at counteracting microbiome dysbiosis caused by non-microbial food contaminants.

## 4. AI Applications in the Interaction of Microplastics and Gut Microbiota: Case Study

A study analyzing 1352 gut microbiota samples from six different animal species systematically explored the impact of microplastic exposure on gut microbiota diversity and function using AI technology [98]. The study employed ML models such as RF, XGBoost, and Artificial Neural Networks (ANNs) for regression analysis. By evaluating the model fitting performance, the team selected the best model to predict the effects of microplastic exposure on gut microbiota diversity (α-diversity, Shannon Index) and community composition (β-diversity). The results demonstrated that exposure duration and particle size were the most important factors affecting microbiota diversity. Different microplastic sizes and types had varying effects on community stability and function, indicating that microplastics, as external environmental stressors, can disrupt the gut ecosystem when exposure is prolonged or occurs at high doses. To further identify key microbial populations that are particularly sensitive to microplastic exposure, this study used classification models to divide the samples into microplastic-exposed and non-exposed groups. The team trained the models with RF, XGBoost, SVM, and ANN, and validated the classification results. The models showed that RF and XGBoost could effectively differentiate microbiota differences between the exposed and non-exposed groups, achieving high classification accuracy and AUC values. These models successfully identified potential biomarkers such as *Lactobacillus*, *Helicobacter*, and *Pseudomonas* (involved in environmental degradation and pollutant transformation), which could effectively distinguish between the exposed and non-exposed groups. To further understand the importance of each variable in the prediction, the team utilized SHAP and Permutation Importance methods. SHAP analysis indicated that exposure duration and microplastic particle size played key roles in predicting microbiota diversity and function. Permutation Importance analysis revealed that environmental factors, such as temperature, also influenced the effects of microplastic exposure. Notably, an interaction effect was observed between temperature and exposure, particularly for pathogenic microbiota and degrading microbiota, suggesting that environmental factors and pollutant exposure can interact to shape gut microbiota composition. Overall, the study demonstrated the substantial potential of AI in the field of foodborne risk assessment, particularly in understanding the effects of microplastic exposure. ML models improved the efficiency and accuracy of evaluating exposure impacts, identifying biomarkers, uncovering key influencing factors, and assessing health risks. The use of multiple models and result interpretation provides valuable insights into the complex relationship between non-microbial food contaminants and the gut microbiome, supporting the development of early risk warning systems.

## 5. Challenges and Perspectives

**Figure 3:**
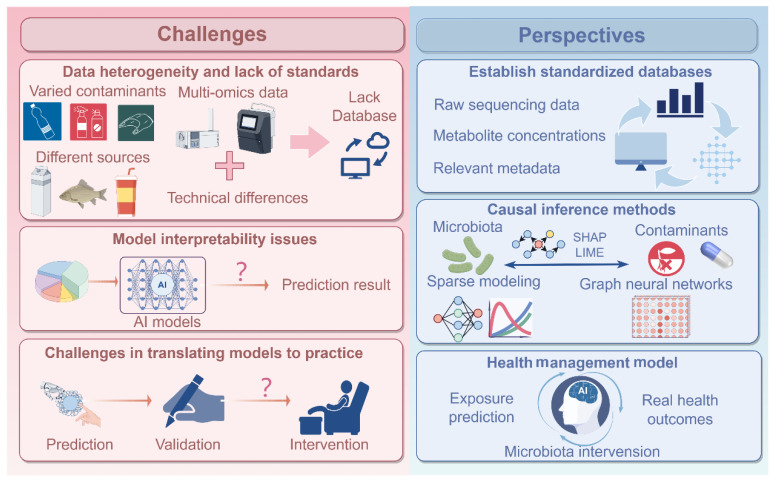
Challenges and perspectives in AI application for the interaction of food contaminants and gut microbiota-mediated health.

Currently, significant progress has been made in the application of AI to study the impact of non-microbial food contaminants on gut microbiota. However, several challenges still remain, which hinder the reproducibility, interpretability, and practical application of research findings in public health (Figure 3).

### 5.1. Data Heterogeneity and Lack of Standards

A primary challenge in research on the impact of non-microbial food contaminants on gut microbiota is the heterogeneity of the data used. Samples used to study the interaction between gut microbiota and environmental exposure are derived from different food matrices, such as blood, urine, feces and varied food samples, and include contaminants that vary widely in nature—pesticides, microplastics, heavy metals, and persistent organic pollutants. The diversity of these contaminants, combined with differences in sample preservation methods, extraction techniques, and sequencing platforms (e.g., Illumina, PacBio, Oxford Nanopore), introduces significant biases that make data comparison and model generalization difficult. Additionally, the use of various databases (e.g., Greengenes, SILVA, GTDB) and sequencing techniques (e.g., 16S rRNA sequencing, metagenomics, metatranscriptomics) can lead to inconsistencies, complicating the integration of data across studies. The challenge extends beyond microbiome composition to other multi-omics data, such as metabolomics, transcriptomics, and exposomics, where variations in measurement techniques (e.g., mass spectrometry, UHPLC-MS/MS, ICP-MS) further contribute to data heterogeneity. For AI models, integrating these diverse data types is essential for drawing meaningful conclusions about the pollutant–microbiome–health relationship. The lack of standardized platforms for data sharing, quality control, and metadata inclusion hinders the development of AI models that can be applied across studies.

To address these challenges, it is crucial to standardize and integrate raw sequencing data, metabolite concentrations, and relevant metadata. Standardization methods, such as z-score normalization for metabolites and relative abundance normalization for microbiome data, ensure comparability across datasets. Key features are extracted from each data type and then combined into a unified dataset. The establishment of standardized multi-omics platforms for non-microbial food contaminants and microbiome data is also essential. A global or regional open database, similar to the International Cancer Genome Consortium (ICGC) and the Human Microbiome Project (HMP), could support AI algorithms by providing high-quality, comparable datasets. This would enable cross-study model training and improve the generalization of predictions. Such a database should include raw sequencing data, metabolite concentrations, and relevant metadata, allowing for more comprehensive analysis and integration across diverse datasets (Figure 3). By incorporating these key data types, the platform would deepen our understanding of the interactions between food contaminants, the microbiome, and host health, facilitating the development of more robust predictive models and targeted interventions.

### 5.2. Model Interpretability Issues

In addition to data heterogeneity, the lack of model interpretability is another critical issue. Many AI models, such as deep neural networks and gradient boosting trees, offer high prediction accuracy but lack clear explanations for how conclusions are drawn, leading to their characterization as “black boxes”. In toxicology and public health research, it is essential not only to predict outcomes but also to understand the underlying causes. For example, some AI models can identify high and low exposure groups, but it is challenging to pinpoint which bacterial genera, metabolic pathways, or microbiota functions contribute to the observed differences. Without these insights, it is difficult to propose testable biological hypotheses or design targeted interventions.

To improve model interpretability, AI technologies like SHAP and LIME can be applied to assess the importance of individual microbiome features in contaminant-related outcomes. For instance, *Lactobacillus* and *Bacteroides* species may show strong associations with organophosphate exposure [98]. However, microbiome data often suffer from issues such as sparsity and collinearity, with strong correlations between the abundance of different genera, leading to model instability. To address this, feature stability selection, sparse modeling (e.g., Lasso, ElasticNet), and causal inference methods should be combined to better differentiate between correlation and causality. In the future, techniques like GNNs could help visualize the complex ecological networks of the microbiome, providing a more interpretable framework for understanding microbiome–contaminant interactions (Figure 3).

### 5.3. Challenges in Translating Models into Practice

Despite many meaningful predictive findings from AI research, translating models into public health policies or clinical interventions remains a significant challenge. Most studies currently stop at drawing predictions from literature-based data, without a clear path from prediction to health management. For example, AI models may categorize high-risk individuals based on their previous behaviors or exposure histories, but these predictions have not yet led to individualized intervention strategies, and there is little clinical validation of whether these interventions would achieve the desired effects.

To bridge the gap between prediction and intervention, the development of AI-based foodborne health management models is essential. Such models should follow a three-step approach: first, exposure prediction to identify high-risk populations and changes in microbiota linked to exposure; second, validation of predictions by comparing them with real health outcomes such as inflammatory markers, immune function, or metabolic abnormalities; and third, using the model to identify sensitive microbiota or metabolic pathways for targeted personalized interventions (Figure 3). This approach will necessitate further mechanistic research to ensure the models’ practical effectiveness and clinical applicability.

## 6. Conclusions

In conclusion, this review provides a comprehensive overview of the impact of non-microbial food contaminants on gut microbiota and the subsequent effects on host health, with a focus on the application of AI in understanding these interactions. Non-microbial food contaminants, such as microplastics, heavy metals, pesticides, antibiotic and veterinary drug residues, and persistent organic pollutants, have been shown to disrupt the balance of the gut microbiota, which plays a key role in regulating metabolism and overall health. AI, particularly ML and DL models, has proven invaluable in predicting and interpreting how these contaminants interact with the microbiome and affect health outcomes. AI has enabled the identification of microbial biomarkers, revealed toxicity mechanisms, and facilitated the development of personalized interventions to mitigate the harmful effects of these contaminants. While challenges such as data heterogeneity and model interpretability remain, advancements in data standardization and AI transparency will enhance the broader application of these tools. The continued development of AI-driven approaches holds significant promise for addressing the health risks associated with non-microbial food contaminants, ultimately improving global food safety.

## Data Availability

No new data were created or analyzed in this study. Data sharing is not applicable to this article.
